# Fatal pulmonary infection with respiratory syncytial virus in an immunocompromised adult patient: A case report

**DOI:** 10.1097/MD.0000000000011528

**Published:** 2018-07-20

**Authors:** Qi Wang, Wei Li, Danhua Qu, Tong Xin, Peng Gao

**Affiliations:** Department of Respiratory and Critical Care Medicine, The Second Affiliated Hospital of Jilin University, Changchun, Jilin, China.

**Keywords:** adult, ground-glass opacity, immunosuppression, respiratory syncytial virus

## Abstract

**Rationale:**

Respiratory syncytial virus (RSV) is a single-stranded negative-sense RNA virus that belongs to the family of paramyxoviruses. RSV is the most common pathogen that causes acute lower respiratory tract infection in infants and young children. However, its incidence in immunocompromised adults remains unclear. In the present study, we report an adult patient with chronic nephropathy, who received long-term immunosuppressants and died of rapid respiratory failure due to RSV infection.

**Patient concerns:**

A 54-year-old male patient with chronic nephropathy, who received long-term immunosuppressants, was admitted to the Department of Respiratory Medicine due to the symptoms of fever, cough, expectoration, and dyspnea.

**Diagnoses:**

Pulmonary radiology revealed multiple bilateral ground-glass opacity. Laboratory tests revealed elevated inflammation indicators, implying infection with bacteria, viruses, and/or fungi. Furthermore, the patient was positive for RSV antibodies, without positive results for other pathogens. Moreover, the patient was immunocompromised due to the long-term use of corticosteroids and immunosuppressants, as evidenced by decreased total IgG levels and reduced CD4 and CD8 T-lymphocyte counts.

**Interventions and outcome:**

Despite the intensive anti-infection treatment and respiratory support, the patient developed rapid progression, and subsequently died of respiratory failure.

**Lessons:**

RSV infection should be fully considered in adults who are immunocompromised or have underlying diseases, such as nephropathy patients receiving long-term immunosuppressants, especially in the presence of respiratory symptoms and computed tomography (CT) chest findings of diffuse ground-glass opacities.

## 1 Introduction

Respiratory syncytial virus (RSV) is the most common pathogen that induces acute lower respiratory tract infection in infants, and has caused approximately 66,000 to 199,000 child deaths worldwide in 2005.^[1]^ It is a single-stranded negative-sense RNA virus that belongs to the family of paramyxoviruses. These viruses transmit through respiratory droplets and intimate contact. The seasonal trends for RSV suggest that infection occurs throughout the year, with the peak in winter, followed by spring and autumn. The typical histological finding of RSV pneumonia is the interstitial infiltration of mononuclear cells, which mainly manifests as the widening of the alveolar space and interstitial monocyte-predominant exudates. Specific diagnosis depends on the isolation of the virus and the demonstration of a serological response, such as serum complement fixation assay and neutralization assay.

RSV is not only a major cause of severe lower respiratory tract infection in young children, but also in the elderly and in severely immunocompromised individuals, which incurs serious complications, respiratory failure, prolonged hospital stay, and even high mortality.^[2]^ The immunity generated after RSV infection is transient, and does not provide long-lasting protection, allowing re-infection even with the same strain of RSV.^[3]^ During RSV infection, humoral immunity to viral exposure plays an important role in the identification of viral surface proteins, and cellular immunity helps the body recognize the virus and infected cells. As an impairment of cellular and humoral immunity in immunocompromised patients, those patients are more susceptible to RSV.^[4]^

However, rare cases of RSV infection that cause adult lower respiratory tract infection, or even mortality, have been reported. In the present study, we present a 54-year-old patient, who was receiving immunosuppressants and admitted to a hospital with symptoms of fever, cough, expectoration, and dyspnea, and was subsequently diagnosed with RSV infection. This patient eventually died, emphasizing the precaution of RSV infection in immunocompromised adults, especially in presence of respiratory symptoms and chest computed tomography (CT) findings of diffuse ground-glass opacities of the lung.

## 2 Case presentation

**Figure 1:**
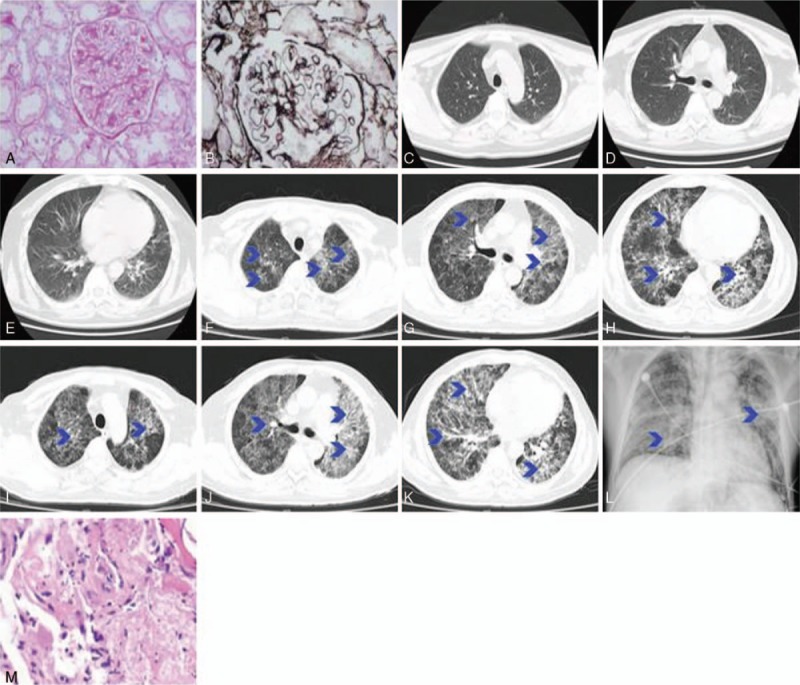
(A) Histological analysis for renal biopsy (H&E staining) revealed focal segmental glomerulosclerosis with mild mesangial cell hyperplasia, endothelial cell proliferation and thickening of the glomerular capillary walls, but with open glomerular tuft. Focal tubular epithelial cell atrophy was observed. (B) Periodic acid methenamine–Masson staining revealed diffuse, segmental lesions as visible mesangial deposition, without “tram-track" appearance. (C–E) Chest CT scan at one month before admission revealed normal findings. (F–H) Chest CT scan on admission revealed bilaterally diffuse ground-glass opacities in the lungs. (I–K) Chest CT scan at the 10th day after admission indicated disease progression. (L) Chest x-ray at 15th days after admission revealed extensive diffuse ground-glass opacification, which worsened compared to that of before. (M) Bronchoscopy pathology revealed pulmonary interstitial fibrosis, hyperplasia of the alveolar epithelium and necrosis in the alveolar cavity.

A 54-year-old male who had a medical history of membranous nephropathy II with nephrotic syndrome (Fig. 1A and B) was administered with long-term oral glucocorticoids and immunosuppressants. The patient had a 20 pack-year history of smoking, and denied a family history of hereditary diseases. Chest x-ray demonstrated normal findings at one month before admission (Fig. 1C–E). On August 8, 2016, the patient was hospitalized for fever accompanied by progressive dyspnea, cough, and expectoration for 5 days. On admission, the BMI of the patient was 24.5 kg/m^2^, and his body temperature was 39.0°C. Furthermore, the patient had symptoms of tachypnea (35 bpm) and severe hypoxemia (SaO_2_ 86%). On auscultation, the patient had good air entrance bilaterally with scattered diffuse crackles and rhonchi. Furthermore, the chest CT scan revealed multiple ground-glass opacities (Fig. 1F–H), and laboratory tests (Table 1) revealed normal white blood cell (WBC) count, but with elevated neutrophil count, C-reactive protein (CRP), erythrocyte sedimentation rate (ESR), and (1→3)-β-D-glucan (Table 2). The patient was diagnosed as RSV infection on the fourth day of hospitalization when positive RSV-Ab was detected.

**Table 1:**
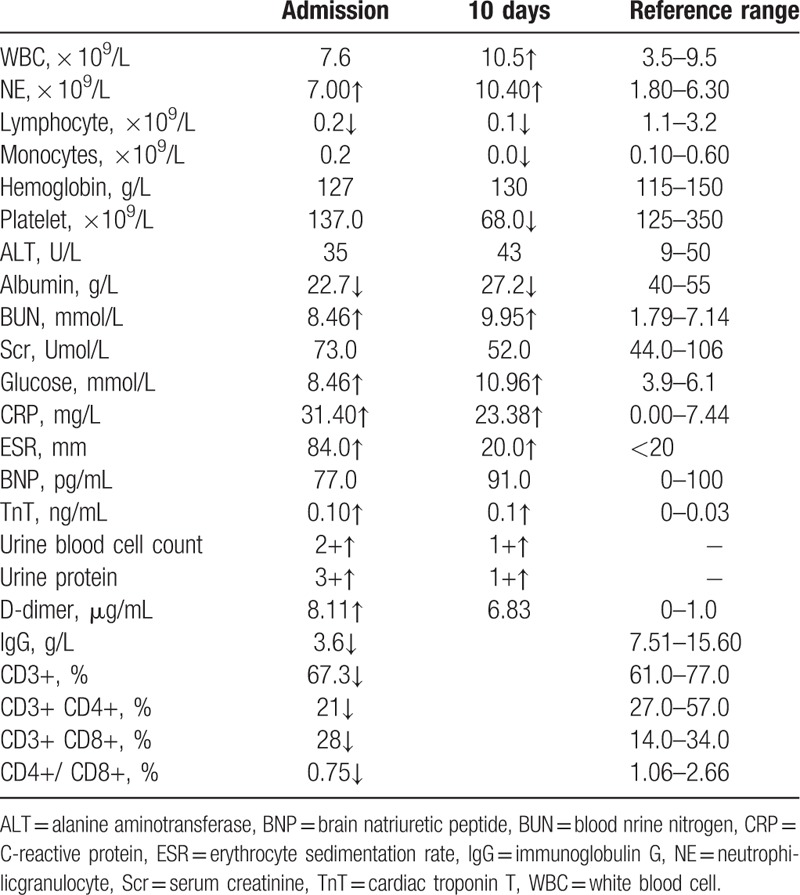
Results of the laboratory tests.

**Table 2:**
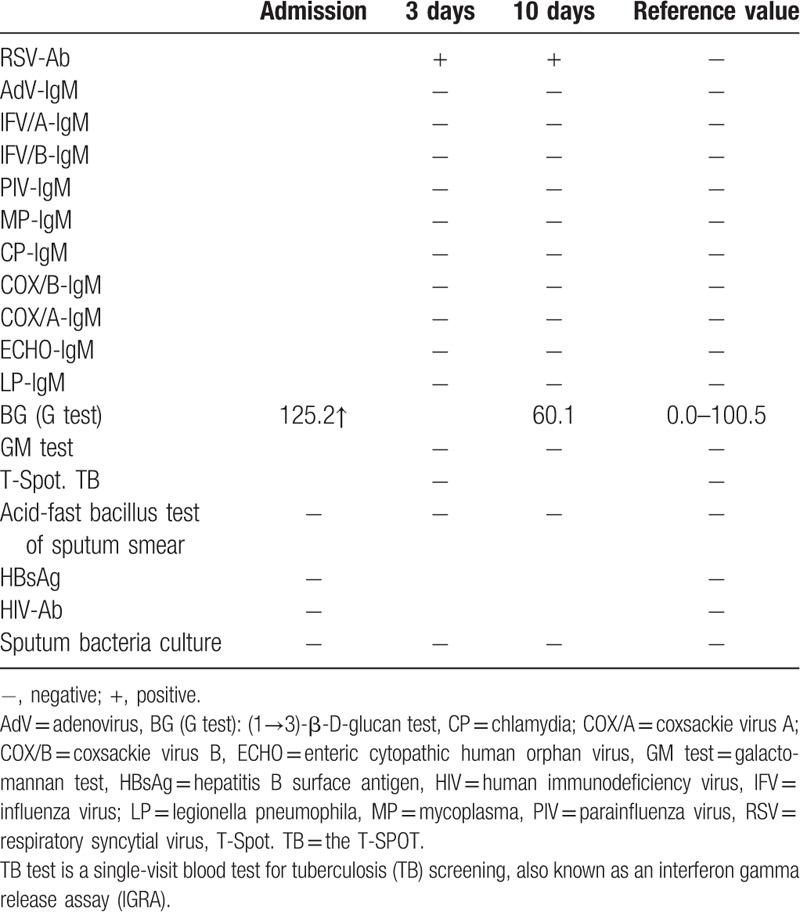
Etiological tests.

On admission, the patient was immediately given respiratory monitoring and supplemental oxygen to improve the low oxygen saturation, as well as antibiotics (moxifloxacin for 4 days, followed by cefminoxine for 8 days), and antifungal therapy (voriconazole for 10 days). The dose of the glucocorticoids and immunosuppressants remained largely unchanged (Table 3). After 10 days of treatment, the patient's condition became worse. Chest CT revealed the progression of the disease (Fig. 1I–K), and oxygen partial pressure was further decreased (Table 4). The patient was transferred to the Emergency Intensive Care Unit, where the patient was intensively treated, including noninvasive mechanical ventilation, broad-spectrum antibiotics (i.v. meropenem, oral moxifloxacin, and cotrimoxazole), antifungal therapy (micafungin), corticosteroids (methylprednisolone 40 mg *bid iv*) to relieve the inflammation, and other supportive treatment. Ganciclovir was also prescribed due to a possibility of viral infection, such as cytomegalovirus. Five days later, the patient's condition was further aggravated based on the chest x-ray evaluation (Fig. 1L). Despite receiving another round of treatments, including invasive ventilator-assisted ventilation therapy, methylprednisolone (80 mg *bid*), antibacterial agents (cefoperazone sulbactam, tigecycline, and cotrimoxazole) and antifungal (micafungin) therapy, the patient eventually died after 2 days.

**Table 3:**
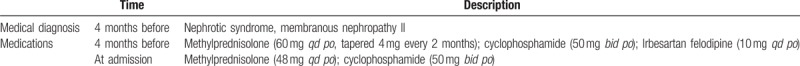
Medical history and medications.

**Table 4:**
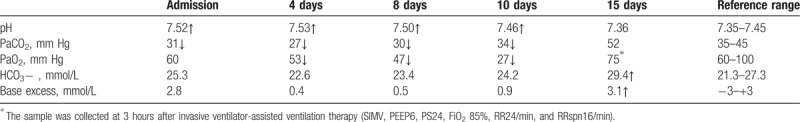
Arterial blood gas analysis.

## 3 Discussion

**Figure 2:**
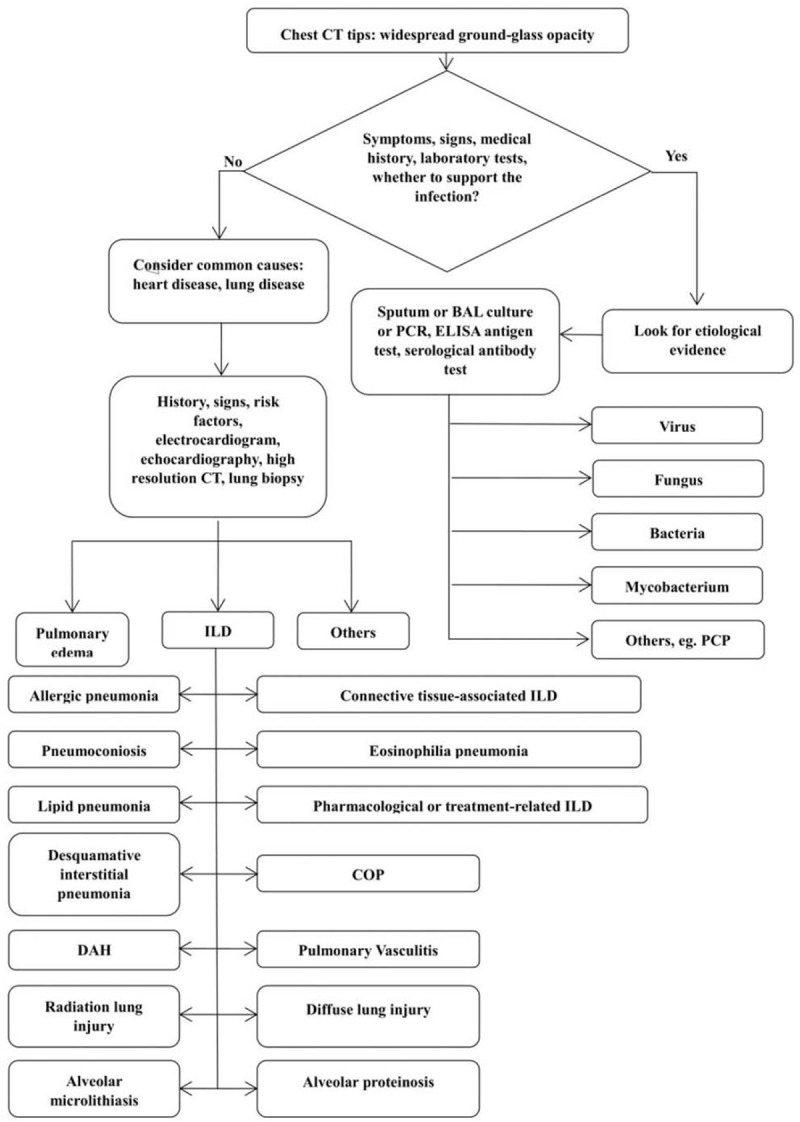
The clinical process for the diagnosis of widespread ground-glass opacity. BAL = bronchoalceolar lavage, COP = cryptogenic organizing pneumonia, DAH = diffuse alveolar haemorrhage, ELISA = enzyme linked immunosorbent assay, ILD = interstitial lung disease, PCP = pneumocystis carinii pneumonia, PCR = polymerase chain reaction.

Ground-glass opacification refers to an area of increased attenuation in the lungs on CT, which result from the partial filling of air spaces in the lungs by exudates, and is probably accompanied by interstitial thickening or partial collapse of the lung alveoli. This specific image indicates some non-infectious diseases, such as pulmonary vasculitis, eosinophilic pneumonia and radiation-induced lung injury (listed in detail in Fig. 2), and infectious diseases. Pathogens that cause ground-glass opacities on CT images include bacteria (*Pseudomonas aeruginosa*, *Staphylococcus aureus*, etc.), fungi (*Aspergillus*, *Mucormycosis*, and *Candida*), viruses (cytomegalovirus, varicella zoster virus, herpes simplex virus, parainfluenza, influenza, etc.), mycobacterium (tuberculosis and nontuberculous mycobacteria) and others (e.g., pneumocystis carinii pneumonia). A previous study revealed that hydrostatic pulmonary edema was the most common cause (56%) for ground-glass opacities on CT images, followed by interstitial lung diseases, and infection accounted for 5%.^[5]^ In particular, diffuse infection (24%) was the second most common cause in immunocompromised patients. In another study, it was shown that the diagnosis of infectious pneumonia mostly caused by pneumocystis, cytomegalovirus, and RSV is common in non-HIV immunosuppressed patients, representing 44% of cases.^[6]^

In the present case, the patient was receiving immunosuppressants and admitted to our hospital with symptoms of fever, cough, expectoration, and dyspnea. Laboratory tests revealed elevated inflammation indicators and positive RSV-Ab, implying possible infection with bacteria, viruses, and/or fungi. The long-term use of immunosuppressive agents led to the abnormal condition of the immune system, in which cellular or humoral immunity was inadequate, and the resistance to infection decreased, allowing opportunistic infections caused by pathogenic bacteria, fungi and viruses. In response to invading microbes, an acute inflammatory response was mounted with the release of pro-inflammatory mediators and the recruitment of inflammatory cells into the lungs, which resulted to increased vascular permeability and pulmonary edema.^[7]^ Immunocompromised patients are at risk for infection with severe acute respiratory manifestation from a variety of opportunistic viral pathogens.^[8–10]^ RSV infection in adults is often asymptomatic, but causes severe lower respiratory tract infection in adults who are immunocompromised, older, and/or have underlying cardiopulmonary diseases.^[11]^ In the present study, the diagnosis of RSV infection was made on the following basis: the patient might have been immunocompromised due to the long-term use of corticosteroid immunosuppressive therapy, as evidenced by the decrease in total IgG level and reduced CD4+ and CD8+ T-lymphocyte count; the illness developed in the epidemic season of RSV; the patient had radiologic imaging changes, in which the initial performance of diffuse ground-glass opacities was accompanied by interstitial fibrosis, and this subsequently gradually developed into pulmonary fibrosis and traction bronchiectasis with a small amount of inflammatory exudates; the patient presented with positive RSV-Ab without positive results for other pathogens. Considering the impairment of immunity in the patient, empiric broad-spectrum antibiotics were used to ensure coverage of both typical and atypical organisms. The determination of serum (1→3)-β-D-glucan level is a noninvasive assay for circulating fungal cell wall components, which allows for the rapid identification of fungal infections.^[12]^ Fungal pneumonia is increasingly common, particularly in highly immunosuppressed patients, and the measurement of serum (1→3)-β-D-glucan level has emerged as an adjunct method for the diagnosis of invasive fungal infections.^[13,14]^ In the present case report, the patient presented with elevated (1→3)-β-D-glucan levels, and was treated with adequate anti-fungal therapy. Despite the intensive anti-infection treatment, the patient developed rapid progression of respiratory failure.

Given RSV infection rarely caused diffuse ground-glass opacities of the lung on chest CT images, highly pathogenic viruses such as cytomegalovirus and adenovirus, rather than RSV, were initially considered, until positive RSV-Ab was detected. This led to a failure to early diagnose and treat RSV infection. The patient experienced rapid deterioration and mortality even under efficient treatment. Lessons should be learned from this case that RSV infection should be fully considered in adults who are immunocompromised or have underlying diseases, such as nephropathy patients on long-term immunosuppressants, especially in the presence of respiratory symptoms and CT chest findings of diffuse ground-glass opacities of the lungs.

## Author contributions

QW and WL carried out the data collection, literature review and drafting of the manuscript. DQ contributed to the drafting of the manuscript and literature review. TX participated in the data collection and drafting of the manuscript. PG helped to draft the manuscript and revised the final version of the manuscript. All authors read and approved the final manuscript.

**Data curation:** Tong Xin.

**Investigation:** Danhua Qu.

**Resources:** Tong Xin.

**Supervision:** Peng Gao.

**Writing – original draft:** Wei Li.
